# Things We Do for No Reason™: Failing to consider primary aldosteronism in the initial evaluation of hypertension, hypertensive urgency, and hypertensive emergency

**DOI:** 10.1002/jhm.70035

**Published:** 2025-03-20

**Authors:** Michael C. Shih, Tessa R. Lavorgna, Prerna Dogra, Christina N. Hirner, Kristen Payne

**Affiliations:** ^1^ Deming Department of Medicine, Section of General Internal Medicine & Geriatrics Tulane University School of Medicine New Orleans Louisiana USA; ^2^ Department of Internal Medicine University of Miami Health System Miami Florida USA; ^3^ Department of Head and Neck‐Endocrine Oncology Moffitt Cancer Center Tampa Florida USA

## Abstract

Hypertension is frequently treated as essential hypertension. However, secondary causes of hypertension should be considered, because distinct treatments are used for different causes of hypertension. Primary aldosteronism is considered a candidate for the most common cause of secondary hypertension. Despite the effects of many antihypertensive agents on the renin–angiotensin–aldosterone system, ongoing efforts to manage hypertension should not be discontinued solely for the purposes of screening. If a patient presents with new or untreated hypertension, screening should be considered before starting antihypertensive agents that could affect the renin–angiotensin–aldosterone system.

## Visual Abstract

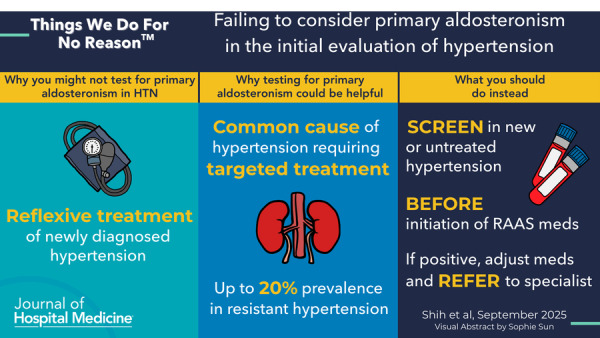

## A CLINICAL SCENARIO

A woman in her mid‐30s presented for shortness of breath since awakening. She additionally had palpitations, and a feeling of chest pressure that radiated to her back. In the emergency room, her blood pressure reached a peak of 190/139 mmHg. She was diagnosed with hypertensive emergency and started on intravenous and oral metoprolol tartrate, which resolved symptoms.

In the emergency department, labs were significant for hypokalemia (3.2 mEq/L), hypomagnesemia (1.7 mg/dL), normal serum bicarbonate (23 mEq/L), 2+ urine protein, and 3+ urine blood. At her most recent clinic visit 3 months ago, her blood pressure was within normal limits. On hospitalist team assessment, the patient's blood pressure had improved to 154/97 mmHg. The hospitalist team thereafter diagnosed the patient with essential hypertension and continued treatment with metoprolol tartrate.

## BACKGROUND

Worldwide, hypertension is considered the most common preventable risk factor for cardiovascular disease, chronic kidney disease, and cognitive impairment.^1^, ^2^ Among the secondary causes of hypertension, primary aldosteronism is commonly considered a leading candidate, with studies showing a prevalence as high as 20% of hypertensive people depending on the population sampled.^3^, ^4^ Few publications specifically examine hospitalized patients. One study found a prevalence of 3.31% for hospitalized patients who had hypertension.^5^ Another study found a prevalence of 2.5% of patients with hypertensive urgency or hypertensive emergency.^6^ Several retrospective studies demonstrate that the screening rates for primary aldosteronism are significantly discordant to its high prevalence. Only 1.3%–2.1% of patients with resistant hypertension are screened for primary aldosteronism.^7^, ^8^, ^9^, ^10^ Furthermore, Ruhle et al. found that only 2.7% of patients with hypertension and hypokalemia were screened.^11^

## WHY YOU MIGHT THINK IT IS APPROPRIATE TO MANAGE HYPERTENSION WITHOUT CONSIDERATION FOR PRIMARY ALDOSTERONISM

Given the frequency with which clinicians encounter hypertension and its complications, it can be habitual to initiate therapeutic interventions without considering that hypertension may be secondary to another pathophysiologic process. For hospitalists, the inpatient setting may further discourage screening of primary aldosteronism. Because intensive blood pressure management in hospitalized adults is associated with greater risk of adverse events, hospitalists may not be inclined to consider additional workup or treatment of hypertension.^12^ Moreover, hypertension due to primary aldosteronism could respond at least partially to common first‐line antihypertensive agents, thereby inducing a false sense of security. Primary aldosteronism screening aims to identify renin‐independent aldosterone overproduction. Multiple antihypertensive medications (angiotensin‐converting enzyme inhibitors, angiotensin receptor blockers, beta‐blockers, diuretics) that frequently serve as first‐line agents for essential hypertension interact with the renin–angiotensin–aldosterone system (RAAS). Providers could therefore be concerned that use of these medications could potentially confound the diagnostic evaluation of primary aldosteronism.^13^

## WHY MANAGING HYPERTENSION WITHOUT CONSIDERING PRIMARY ALDOSTERONISM IS NOT HELPFUL

Various causes of hypertension (i.e., secondary hypertension) have distinct treatments, and indiscriminately treating as essential hypertension could lead to a delay in appropriate diagnosis and treatment.^14^ In a survey study of patients with primary aldosteronism, approximately 30% of patients were found to have primary aldosteronism 1–3 years after they were diagnosed with hypertension; over half of patients had a delay of greater than 3 years.^15^ A retrospective study found that half of patients with hypokalemic primary aldosteronism experienced delays in diagnosis, with a median delay of 4.5 years. Missed diagnosis and delayed treatment of primary aldosteronism is particularly dangerous, as patients with primary aldosteronism are at higher risk for cardiovascular events and renal dysfunction compared to those with essential hypertension at similar blood pressure levels.^16^, ^17^ This heightened risk is attributed to aldosterone‐mediated end‐organ damage resulting in endothelial dysfunction, vascular remodeling, fibrosis, and oxidative stress.^18^ Hundemer et al. found that compared to patients with essential hypertension, those with suppressed renin activity had excess risk for cardiovascular events and mortality with hazard ratio (HR) 2.83. Those who were adequately treated with unsuppressed renin had no significant excess risk.^19^

Effective therapies for primary aldosteronism differ from those of essential hypertension. For example, primary aldosteronism due to bilateral disease is treated with mineralocorticoid receptor antagonists (e.g., spironolactone or eplerenone), which are not typical first‐line agents for management of essential hypertension. Similarly, primary aldosteronism due to unilateral disease can be effectively managed with adrenalectomy, which would not be appropriate for essential hypertension. Timely diagnosis allows for targeted medical and/or surgical management, which can help patients achieve blood pressure goals, reduce risk for cardiovascular events, reduce left ventricular mass, improve hypokalemia, improve kidney function, and reduce risk for all‐cause mortality.^20^, ^21^, ^22^, ^23^, ^24^, ^25^, ^26^

## WHAT YOU SHOULD DO INSTEAD OF PRESUMPTIVELY TREATING HYPERTENSION AS ESSENTIAL HYPERTENSION

To detect primary aldosteronism, providers must first consider it as a possibility. The 2016 Endocrine Society guidelines recommend screening for primary aldosteronism in hypertensive patients with any of the following:

(1) Spontaneous or diuretic‐induced hypokalemia.
(2) Hypertension resistant to three conventional antihypertensive drugs (including a diuretic).
(3) Controlled blood pressure (<140/90) on four or more antihypertensive drugs.
(4) Sustained blood pressure above 150/100 on three separate measurements on different days.
(5) A mass on the adrenal gland (i.e., adrenal incidentaloma).
(6) Sleep apnea.
(7) A family history positive for stroke or hypertension before age 40.
(8) First‐degree relatives with primary aldosteronism.

Historically, clinicians often believed that a 4 week “washout” of antihypertensive medications was necessary to achieve accurate primary aldosteronism screening results. However, the benefits of optimizing primary aldosteronism testing need to be weighed against the detriments of discontinuing antihypertensive treatment or replacement with less efficacious medications. From a clinical perspective, this step is not necessary in most scenarios. Liu et al. found that primary aldosteronism screening had a 96% sensitivity and 61% specificity even before undergoing a medication washout period. The high sensitivity implies that false negatives are rare in this scenario. Moreover, false‐positive results can be minimized by using a combination of a higher cutoff for plasma aldosterone concentration (PAC) (e.g., ≥10 ng/dL; ≥277 nmol/L) with suppressed plasma renin activity (PRA) (e.g., <1.0 ng/mL/h).^27^, ^28^ However, these cutoffs are not rigid criterion as some medical centers use slightly higher or lower cutoffs.^29^ The plasma aldosterone to renin ratio is denominator dependent (renin) and risks misinterpretation as laboratories have different lower detection limits for PRA, and slight variations in renin can cause significant changes in the ratio.^27^ While it is not standardized, a ratio greater than 30 should be considered for primary aldosteronism. To further increase sensitivity in a hospital setting, lab samples should be collected after correction of hypokalemia.^8^ Moreover, samples should be collected in the morning after the patient has been awake for 2 h and usually seated for 5–15 min.^8^, ^30^ If initial testing is negative and clinical suspicion for primary aldosteronism remains high, then antihypertensive medications can be changed to those with limited effect on RAAS, such as alpha‐1 adrenergic receptor antagonists or hydralazine.^13^ Thereafter testing can be repeated in a few weeks (4 weeks if a mineralocorticoid receptor antagonist or thiazide diuretic was previously used, or 2 weeks for other antihypertensives).^28^

In summary, if suspicion for primary aldosteronism is high enough to warrant screening, ongoing management of hypertension should not be discontinued solely for maximizing screening accuracy.^28^ Instead, the provider should conduct primary aldosteronism screening and interpret the results in the context of the patient's current medications. Conversely, it may be reasonable to withhold treatment or consider the use of antihypertensive medications with minimal RAAS interaction for a short period in those with new asymptomatic and uncomplicated hypertension.

In instances of hypertensive urgency, when symptoms related to hypertensive emergency are absent, screening for primary aldosteronism can occur.^31^ In cases of hypertensive emergency, medications such as verapamil and hydralazine may be useful before obtaining the needed laboratory samples. These practices would improve the integrity of primary aldosteronism screening, thereby guiding patients toward targeted treatment selection and improved health outcomes.

## RECOMMENDATIONS

(1) If primary aldosteronism screening is warranted, and the patient remains asymptomatic and without complications of hypertension, consider withholding a new antihypertensive regimen until blood samples can be obtained.
(2) For patients who are symptomatic (e.g., hypertensive emergency) or exhibit complications of hypertension, do not discontinue ongoing efforts to manage hypertension. Instead, interpret primary aldosterone screening results in the context of medication effects.
(3) Patients should be referred to endocrinology or hypertension specialists like nephrology or cardiology. However, which specialty should preferentially manage primary aldosteronism might differ based on the institution and available resources.

## CONCLUSION

Returning to the clinical scenario, metoprolol tartrate was continued until PAC and PRA could be obtained in the morning. After blood samples were obtained, the patient was transitioned from metoprolol tartrate to lisinopril. The patient's blood pressure improved to 152/116, and nifedipine was added. The patient was discharged with a blood pressure of 134/84.

Resulting 1 week after discharge, PRA (0.168 ng/mL/h) was suppressed (cut‐off PRA < 1.0 ng/mL/h), and PAC (16.9 ng/dL) was significantly elevated (cut‐off PAC > 10 ng/dL). Even when considering that metoprolol tartrate increases the risk for false positive primary aldosteronism screenings, the patient's laboratory results were interpreted as a positive screen.^27^ The patient was referred to a nephrology and hypertension clinic to continue workup for new and complicated hypertension.

## CONFLICT OF INTEREST STATEMENT

The authors declare no conflict of interest.
